# A Comparison Between the Efficacy of Trigeminal Ganglion Radiofrequency Thermocoagulation and Ultrasound-Guided Maxillary-Mandibular Nerve Pulsed Radiofrequency in the Treatment of Trigeminal Neuralgia: A Randomized Clinical Trial

**DOI:** 10.7759/cureus.61565

**Published:** 2024-06-03

**Authors:** Gokhan Yildiz, Omer Taylan Akkaya

**Affiliations:** 1 Pain Clinic, Ankara Etlik City Hospital, Ankara, TUR

**Keywords:** ultrasonography, fluoroscopy, trigeminal ganglion, headache, mandibular nerve, maxillary nerve, radiofrequency ablation, pulsed radiofrequency treatment, trigeminal neuralgia

## Abstract

Background and objective

Trigeminal neuralgia (TN) is a debilitating disorder characterized by acute episodic attacks of pain that significantly impair patients' quality of life and overall functioning. Initial therapeutic strategies to treat this condition include pharmacological options, particularly carbamazepine. In cases with resistance to dose escalation and polypharmacy, interventional procedures may be warranted. The primary aim of this study was to compare the efficacy of trigeminal ganglion (TG) radiofrequency thermocoagulation (RFT) and ultrasound (US)-guided maxillary/mandibular (max/mand) nerve pulsed radiofrequency (PRF) for treating TN, based on the findings at six months post-treatment. The secondary aims were to assess the impact of these interventions on drug consumption and interventional safety based on adverse events.

Methods

This prospective, randomized, single-blind study was conducted at a single pain clinic. Forty-four patients were randomized into two groups. Group RFT received TG RFT at 60 °C, 65 °C, and 70 °C for 60 seconds each, whereas Group PRF received max/mand PRF for 240 seconds. Pain relief was assessed by using the numeric rating scale (NRS) and intervention effectiveness on medication consumption was evaluated by using the Medication Quantification Scale III (MQS III). The rates of intervention-related adverse events were also compared.

Results

Both RFT and PRF significantly alleviated pain at one and six months post-treatment compared to baseline (p<0.05). No statistical differences were found in the NRS and MQS III scores between the groups. At six months, 77.3% of RFT patients and 63.9% of PRF patients experienced at least 50% pain relief, with no statistically significant difference. Hypoesthesia occurred in two RFT patients, and masseter weakness was observed in one patient, while no adverse events were reported in the PRF group.

Conclusions

TG RFT and max/mand PRF are effective treatments for TN. US-guided max/mand PRF, which avoids RFT-associated complications and radiation exposure, may be the superior and preferable option. In this study, the potential space between the coronoid process and maxilla was used to access the maxillary nerve during the maxillary block and PRF procedures, in contrast to the classical approach through the mandibular notch. Further large-scale randomized controlled trials are required to gain deeper insights into the topic.

## Introduction

Trigeminal neuralgia (TN) is defined by the International Headache Society as a disorder characterized by recurrent unilateral brief electric shock-like pain, abrupt onset, and termination, limited to the distribution of one or more divisions of the trigeminal nerve [1]. The rate of incidence of TN is estimated to be approximately 4-29 per 100,000 person-years, with a lifetime prevalence of 0.16%-0.3%. It is more common in women and its incidence increases with age [2]. TN seriously affects the daily lives of patients. While specific data on the rate of labor loss due to TN is not available, studies have indicated that 34% of patients' working lives are affected [3]. Severe pain episodes can significantly affect an individual's ability to work and perform daily activities, potentially leading to significant economic and employment impacts. Therefore, it is extremely important to prevent TN-induced pain attacks that accompany simple daily activities, such as eating, drinking, or touching.

The management of TN is a complex and multifaceted challenge. Initially, conservative management is typically preferred, which includes pharmacological and physical treatments, such as carbamazepine and oxcarbazepine, and physiotherapy [4], which are effective in reducing nerve pain. The first-line pharmacological treatment options - carbamazepine and oxcarbazepine - are known to have 25-30% treatment resistance or produce side effects that patients find intolerable [3]. In such cases, interventional procedures are recommended [5]. Among these, radiofrequency thermocoagulation (RFT) applied to the trigeminal ganglion (TG) is the most commonly utilized modality, especially when surgery, such as microvascular decompression (MVD), is not being considered. RFT is considered an appropriate alternative for patients who are either unable or unwilling to undergo surgical intervention owing to associated risks. It has been recognized for its high success rate in symptom alleviation [6]. However, it is important to note that RFT can lead to serious complications including anesthesia dolorosa, keratitis, aseptic meningitis, hematoma, hypoesthesia, and masseter weakness [7].

RFT involves delivering a low-energy, high-frequency alternating current to biological tissues, causing molecular oscillations and friction that generates heat. This heat leads to coagulation necrosis and tissue destruction, which can alleviate pain by disrupting nerve function [8]. Pulsed radiofrequency (PRF), on the other hand, is a non-destructive method that delivers high-frequency current in short pulses, maintains tissue temperature below the neurodestructive threshold (42 °C), and minimizes heat production. PRF modulates pain signals and gene expression, potentially reducing neuroinflammation and promoting regenerative mechanisms [9]. Interventions targeting the maxillary or mandibular (max/mand) nerve under ultrasound (US) guidance are emerging as viable alternatives to TG RFT, especially in the hands of practitioners experienced with US techniques [10]. This approach can offer effective pain relief while reducing the risk of side effects associated with traditional RFT.

While there is one relevant study [10] in the literature evaluating the efficacy of max/mand nerve block for TN, there is no study on max/mand nerve PRF. In light of this, the primary aim of this study was to compare the efficacy of TG RFT and max/mand nerve PRF for treating TN. The secondary aims were to determine the effects of the interventions on drug consumption and interventional safety based on adverse events. In addition, in this study, we used a different approach than the classic approach between the coronoid process (CoP) and condylar process (CP) of the mandible to access the maxillary nerve under US guidance. The maxillary nerve was accessed by visualizing the maxillary artery (MA) and related structures through the potential gap between the CoP and maxilla anterior to the CoP.

## Materials and methods

Study design and participants

This prospective, randomized, observer-blinded study was conducted in the pain department of a tertiary-care hospital. The approval for this study was granted by the local ethics committee (approval no: AESH-EK1-0001). This trial was registered at ClinicalTrials.gov (registration no: NCT06366139). Before participation, written informed consent was obtained from all patients, ensuring adherence to ethical standards. The study period spanned from January 11, 2023, to March 7, 2024, during which data collection and analysis were systematically carried out.

Patients diagnosed with TN undergo neurological examination by a neurologist and dental examination by a dentist as part of the routine practice of our pain clinic. Further assessments included cranial MRI and MRI angiography to identify any vascular compression of the trigeminal nerve or other secondary causes.

The inclusion criteria were as follows: 1) age >18 years; 2) idiopathic TN based on the International Headache Society criteria; 3) pain for at least six months and a numeric rating scale (NRS) score greater than 6 despite medical treatment (despite the maximum tolerated dose of carbamazepine, gabapentinoid, or tramadol).

The exclusion criteria were as follows: 1) vascular compression of the trigeminal nerve by MRI and MRI angiography and the presence of secondary causes such as multiple sclerosis; 2) trigeminal autonomic cephalalgias accompanied by autonomic symptoms; 3) dental or temporomandibular joint pathologies; 4) previous interventional procedures or surgery for TN; 5) coagulopathy or use of antiaggregants and anticoagulants; 6) cardiac pacemaker; 7) renal-hepatic insufficiency; 8) diagnosis of psychiatric illness; 9) malignancy; and 10) injection site or systemic infection.

Randomization

To ensure the integrity of our study and the comparability of the intervention groups, we employed a computer-assisted randomization method. This process was facilitated using a secure web-based software system designed to generate a random allocation sequence. Participants were assigned unique identifiers upon entry into the study, after which the software randomly allocated them to intervention groups in a 1:1 allocation ratio. The randomization algorithm was designed to be both unpredictable and irreproducible with a built-in mechanism to prevent any form of selection bias. The outcome assessor was blinded to treatment allocation.

Interventions

All procedures were performed under aseptic conditions. All patients were monitored according to the standards of the American Society of Anesthesiologists and an intravenous line was established. A nasal cannula provided oxygen support at 2 L/min. In addition to local anesthesia, all patients received an average of 0.04 mg/kg of midazolam to maintain response to electrical radiofrequency stimulation. All procedures were performed by a single physician with at least five years of experience in ultrasound and fluoroscopy (FL). Patients were followed up in the postoperative care unit for at least one hour after the procedures for possible complications.

Maxillary nerve diagnostic block and pulsed radiofrequency procedure

The patient was placed in the lateral decubitus position with the affected face up. The linear 5-12 mHz US probe (LOGIQ P9, GE Healthcare, Seoul, Korea) was placed below the zygomatic arch at a 45° angle with the horizontal plane. The probe was advanced slightly anteriorly towards the front of the CoP of the mandible. The probe was positioned to view the maxilla anteriorly, the CoP of the mandible posteriorly, and the lateral pterygoid plate (LPP) at the base. In this section, the MA was visualized between the maxilla and LPP using the Doppler function of US. Local anesthesia was administered to the skin and subcutaneous tissues using a 27-gauge needle and 1 mL of 2% lidocaine before needle insertion. Using an out-of-plane approach, a 22-gauge blunt-tipped spinal needle was advanced anterior to the CoP from caudal to cranial, lateral to medial, toward the intersection of the LPP and the maxilla, preserving the artery. Real-time US guidance allowed for direct localization of the MA and identification of the needle tip. In this position, the nerve cannot be visualized directly because of the deep location of the maxillary nerve; however, the MA and nerve are in close proximity.

**Figure 1:**
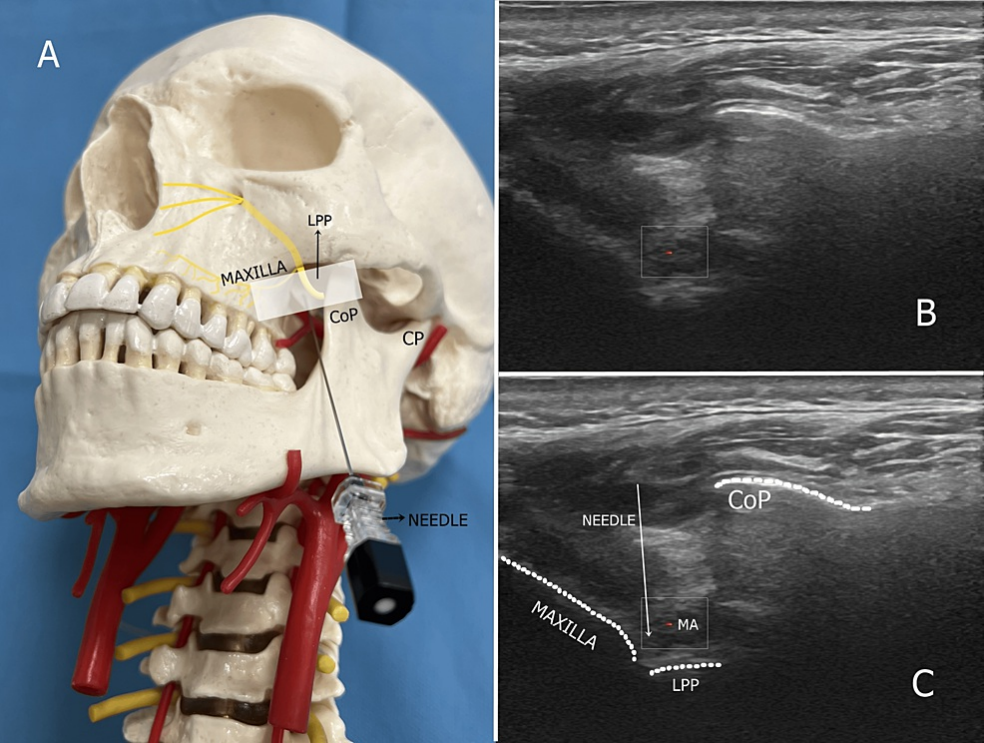
Ultrasound view and probe and needle placement for maxillary nerve and artery Figure 1A shows the placement of the needle and ultrasound probe for the maxillary nerve: the maxilla anteriorly, CoP posteriorly, LPP, and MA at the base. Figure 1B shows the ultrasound view of the maxilla, CoP, LPP, and Doppler image of MA. Figure 1C shows the ultrasound view of the maxilla, CoP, LPP, and Doppler image of the MA, and the direction of the needle CoP: coronoid process; CP: condylar process; LPP: lateral pterygoid plate; MA: maxillary artery

After negative aspiration, 3 mL of a mixture of 0.25% bupivacaine and 1% lidocaine was injected for diagnostic blockade of the maxillary nerve around the artery. Patients with 50% or greater pain reduction for at least four hours after diagnostic blockade underwent maxillary nerve PRF procedure the following day. For PRF application, a 5 mm active type, and 100 mm RF cannula (TOP Nuropole Needle, TOP Corporation, Tokyo, Japan) was advanced using the same technique as the diagnostic block. The maxillary nerve was identified by applying sensory stimulation at a frequency of 50 Hz at a current of 0.5-1 V through an RF generator (TOP Lesion Generator, TOP Corporation) around the artery. After obtaining appropriate responses (fullness, tingling, pressure) in the patient's painful maxillary nerve dermatome, PRF was applied at 42 °C for 240 seconds. Probe placement, the relationship of the MA with bony structures, and US images are shown in Figure 1.

Mandibular nerve diagnostic block and pulsed radiofrequency procedure

For the mandibular nerve, the US probe was advanced posteriorly towards the CP parallel to the zygomatic arch. The patient was asked to open the mouth slightly, and the CoP, condylar process, mandibular notch, and LPP at the base were visualized. Local anesthesia was administered to the skin and subcutaneous tissues using a 27-gauge needle and 1 mL of 2% lidocaine before needle insertion. The needle was advanced using an out-of-plane technique from caudal to cranial and lateral to medial, and the real-time image of the needle tip was taken at a depth of approximately 3-4 cm. The mandibular contraction was obtained with the help of a nerve stimulator for mandibular nerve blockade. After negative aspiration, 3 mL of a mixture of 0.25% bupivacaine and 1% lidocaine was injected for diagnostic blockade of the mandibular nerve. Patients with 50% or greater pain reduction for at least four hours after diagnostic blockade underwent mandibular nerve PRF procedure the following day.

**Figure 2:**
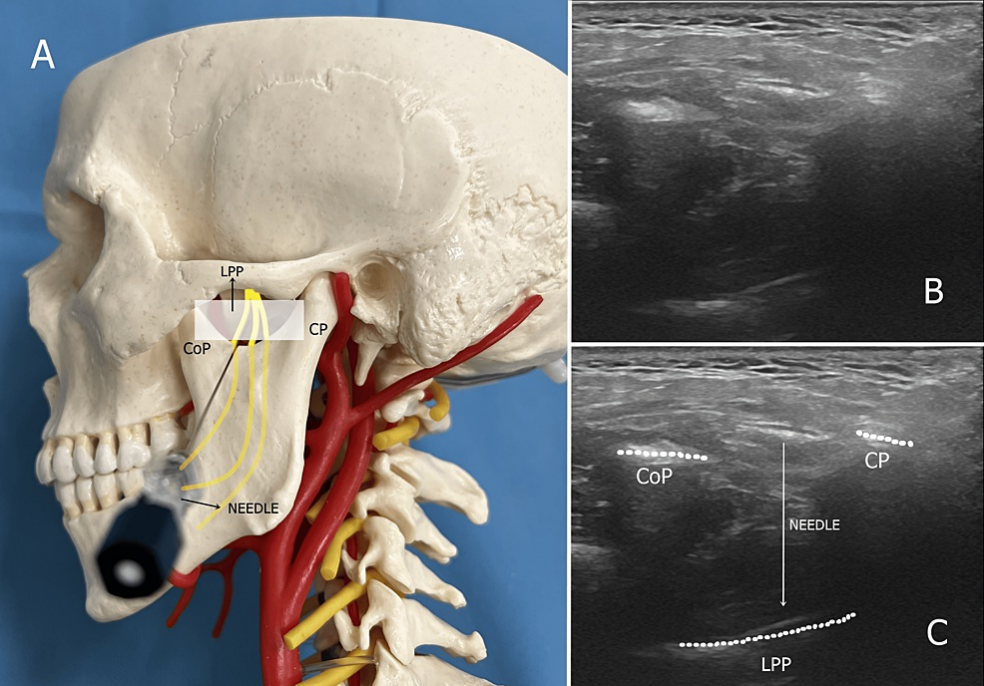
Ultrasound view and probe and needle placement for mandibular nerve Figure 2A shows the placement of the needle and ultrasound probe for the mandibular nerve: the CoP anteriorly, CP posteriorly, and LPP at the base. Figure 2B shows the ultrasound view of the CoP, CP, and LPP. Figure 2C shows the ultrasound view of the CoP, CP, LPP, and direction of the needle CoP: coronoid process; CP: condylar process; LPP: lateral pterygoid plate

For the PRF application, an RF cannula was advanced using the same technique as the diagnostic block. A paresthesia response was sought in the painful mandibular region with sensory stimulation at a current of 0.5-1 V at a frequency of 50 Hz through an RF generator to detect the mandibular nerve. To elicit a contraction response in the muscles of one jaw, motor stimulation was applied at a frequency of 2 Hz and a current of 1-2 V, and a contraction response was observed. After appropriate responses were obtained, PRF was applied at 42 °C for 240 seconds. The probe placement and US images for the mandibular nerve PRF procedure are shown in Figure 2.

Trigeminal ganglion radiofrequency thermocoagulation procedure

The patient was placed in a supine position with the head slightly extended. The head was immobilized softly to prevent head movement of the patient during sedation to prevent any harm. The facial area was sterilized to protect the eye. To locate the foramen ovale (FO), the C-arm FL head was rotated obliquely by approximately 20-30°. The C-arm was then rotated approximately 30-35° in the caudocephalad direction. The submental oblique view showed the FO medial to the mandibular CoP, and the lateral view showed the clivus and skull base. A skin wheal was raised over the shadow of the FO at an entry point 2-3 cm lateral to the commissura labialis (angle of the mouth) with 1 mL of 2% lidocaine, using a 27-gauge needle. Using the tunnel view technique, a 5 mm active type, 100 mm radiofrequency cannula was inserted toward the FO. A lateral view was obtained to determine the position of the needle. The needle was advanced under lateral-view fluoroscopic guidance to a point below the clivus.

**Figure 3:**
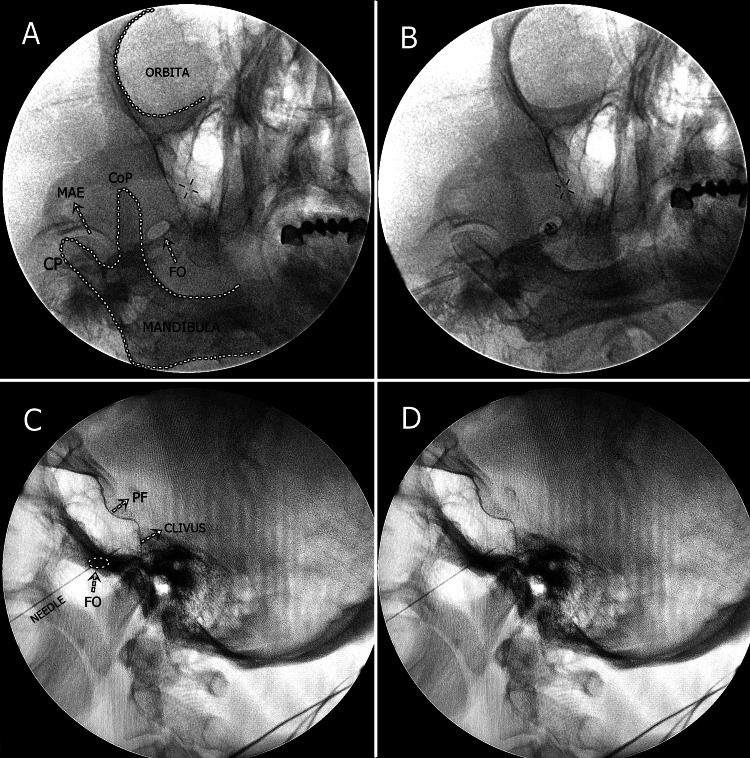
Submental oblique and lateral fluoroscopic images of the foramen ovale Figure 3A shows a submental and oblique fluoroscopic image of the FO just medial to the CoP of the mandible. Figure 3B shows the needle inserted into the FO in submental and oblique fluoroscopic images. Figures 3C-3D show the final position of the needle passing through the FO in the lateral fluoroscopic image FO: foramen ovale; CoP: coronoid process; CP: condylar process; MAE: meatus acusticus externnus; PF: pituitary fossa

The cannula was then connected to the RF generator. Sensory stimulation was applied at a current of 0.1-0.5 volts (V) and a frequency of 50 Hz to elicit a paresthesia response in the corresponding dermatome. A motor response was obtained for the mandibular nerve at a frequency of 2 Hz, which is twice the sensory stimulation current. After sensory and motor responses were obtained, propofol was administered at a dose of 0.5 mg/kg for sedation before the thermal lesion was created. No local anesthetics were administered because of sedation before applying the thermal lesion to the TG. Typically, a 60 s lesion cycle was repeated at 60 °C, 65 °C, and 70 °C. Thereafter, no medication was administered. Submental oblique and lateral fluoroscopic images of the FO with needle insertion and related structures are shown in Figure 3.

Assessment

In this study, the assessment of participants was meticulously conducted at three distinct time points: before the interventional procedures and then at one and six months post-procedure. The assessor, who was blinded to the patients’ identities and the specific interventions administered, had no prior interactions with the participants. This ensured an unbiased assessment of the outcomes. Data on demographic characteristics, specific branches of the trigeminal nerve affected, duration of pain experienced, and medications used for treating TN were meticulously gathered before the intervention. The efficacy of the interventional treatments was measured using the NRS, where a score of 0 indicated no pain and 10 represented the highest pain experienced during the most severe episode. The patients were asked to rate their pain on this scale at each assessment point.

The Medication Quantification Scale III (MQS III) was also employed, but only after six months, to evaluate the effect of the interventions on medication consumption. MQS III is a validated tool in medical research that quantifies medication regimens by assigning a numerical value to each medication based on its class, dosage, and associated risks [11]. This scale aids clinicians and researchers in tracking changes in pain levels throughout a treatment course or study and provides an objective measure of medication consumption and its potential negative impact. Furthermore, adverse events related to the interventions were systematically recorded, providing a comprehensive overview of the efficacy and safety of the interventional treatments administered. A 50% or greater reduction in NRS score was considered clinically significant pain relief.

The primary outcome of this study was treatment response, as measured by the change in NRS score at one and six months post-treatment. The secondary outcomes were the effects of the interventions on medication consumption as assessed by the MQS III score and based on procedure-related adverse events.

Sample size and statistical analysis

The sample size was calculated using G*Power 3.1.9.4 software with an effect size of 1.053, α=0.05, and power (1-β)=0.95. We planned to include a minimum of 21 patients in each group. For this analysis, statistically significant visual analog scale score data at one month (1.33 ± 0.27 for the PRF group and 0.636 ± 0.9 for the RFT group) were used in the study by Elawamy et al. [12].

In this study, all analyses were conducted using the Jamovi Project (version 2.3) software. The study results are reported in terms of frequency and percentage. To assess normality, we employed the Shapiro-Wilk test, examined skewness and kurtosis, and created histograms. For normally distributed variables, we presented the mean and standard deviation (SD). Categorical variables were compared using the chi-square test. To compare the numerical dependent variables between groups, we used independent samples t-tests and Mann-Whitney U tests. For repeated measures, we applied Wilcoxon and Friedman’s tests with Bonferroni correction for multiple t-tests. The threshold for statistical significance was set at p<0.05.

## Results

**Figure 4:**
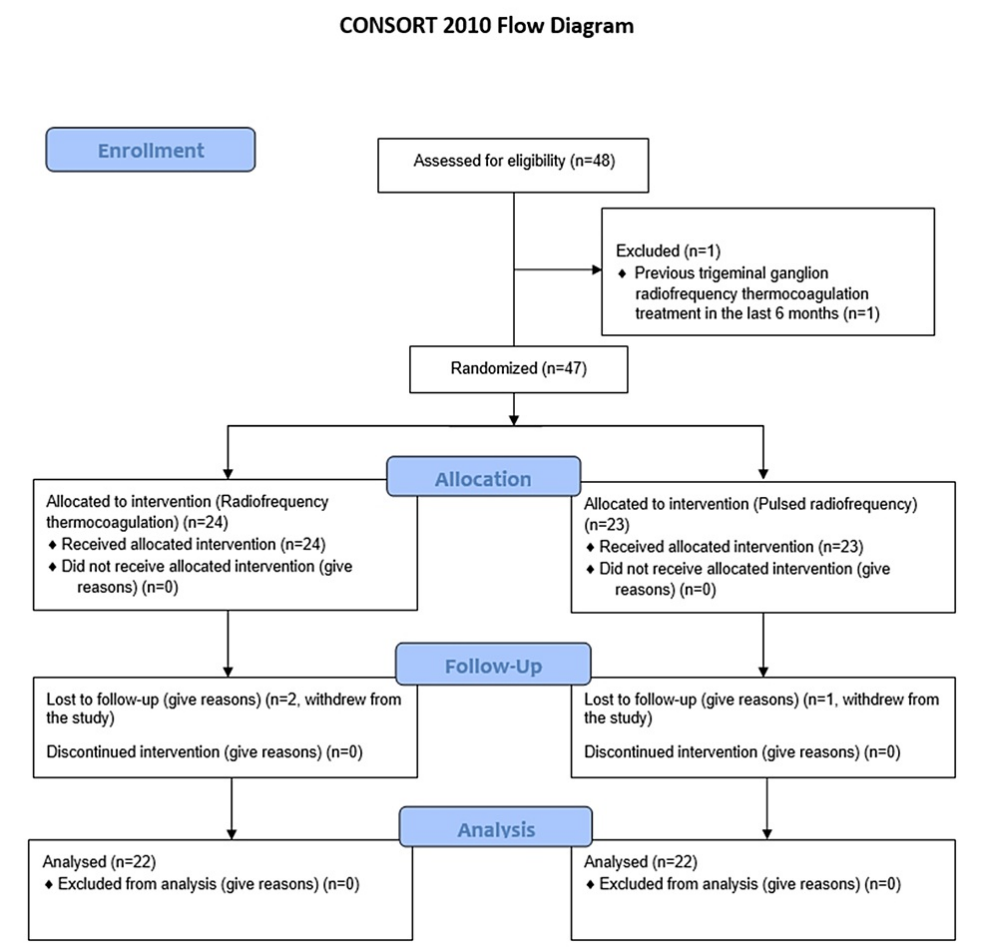
Flow chart depicting patient selection

A total of 48 patients were screened for eligibility. One patient was excluded for having undergone TG RFT six months previously. The remaining 47 patients were randomized into two groups: 24 patients who underwent TG RFT (RFT group) and 23 patients who underwent max/mand nerve PRF (PRF group). Because two patients in the RFT group and one patient in the PRF group were lost to follow-up, the study was completed with 22 patients in each group. The flowchart depicting patient selection is shown in Figure 4.

There were no significant differences between the groups in terms of age, sex, trigeminal nerve branch involvement, or pain duration. Analgesic use before treatment and the change in analgesic consumption at six months after interventions (MQS III) were also similar in both groups (p>0.05). Procedure-related adverse events were hypoesthesia in two patients and masseter weakness in one patient in the RFT group, while no adverse events were observed in the PRF group; however, the difference was not significant (p>0.05). These adverse events resolved spontaneously within one month at the maximum duration (Table 1). According to the six-month evaluation, 17/22 patients (77.3%) in the RFT group had a 50% or greater decrease in NRS score, showing significant pain relief, while this number was 14/22 (63.6%) in the PRF group. This difference was not statistically significant (p>0.05) (Table 1).

**Table 1:** Demographic characteristics and group comparisons ^a^Independent sample t-test; ^b^chi-square test; ^c^Mann-Whitney U test NRS: numeric rating scale; MQS III: Medication Quantification Scale version III; CBZ: carbamazepine; PRG: pregabalin; GBP: gabapentin; TRMD: tramadol; test st: test statistic; SD: standard deviation; MW: masseter weakness; Group RFT: trigeminal ganglion radiofrequency thermocoagulation group; Group PRF: maxillary or mandibular (max/mand) nerve pulsed radiofrequency group

Variables		Group RFT		Group PRF			P-value
		Mean ±SD	Median (range)	Mean ±SD	Median (range)	Test st.	
Age, years		67 ±7.95	66 (52-89)	66.36 ±7.2	66 (54-85)	0.278	0.782^a^
Sex, n (%)	Female	13 (59.1%)		13 (59.1%)			1.000^b^
	Male	9 (40.9%)		9 (40.9%)			
Nerve branch, n (%)	Maxillary	5 (22.7%)		7 (31.8%)			0.795^b^
	Mandibulary	9 (40.9%)		8 (36.4%)			
	Max + mand	8 (36.4%)		7 (31.8%)			
Duration of pain, months		30.64 ±5.62	31.5 (20-40)	31.05 ±5.37	31 (21-41)	-0.247	0.806^a^
NRS baseline		7.91 ±0.75	8 (7-9)	7.82 ±0.73	8 (7-9)	226	0.685^c^
NRS at 1 month		2.73 ±2.64	2 (0-8)	3.55 ±2.61	2.5 (0-9)	289	0.264^c^
NRS at 6 months		3.77 ±2.02	3 (1-8)	4.41 ±2.13	3 (2-9)	274	0.436^c^
Analgesic use, n (%)	CBZ	8 (36.4%)		10 (45.5%)			0.925^b^
	CBZ + PRG	9 (40.9%)		7 (31.8%)			
	CBZ + GBP	3 (13.6%)		3 (13.6%)			
	CBZ + TRMD	2 (9.1%)		2 (9.1%)			
MQS at 6 months		1.78 ±1.37	2.1 (0-4.9)	1.52 ±1.32	2.1 (0-2.8)	231	0.784^c^
Adverse events, n (%)	None	19 (86.4%)		22 (100%)			0.200^b^
	Hypoesthesia	2 (9.1%)		0			
	MW	1 (4.5%)		0			
NRS 50% relief, n (%)	No	5 (22.7%)		8 (36.4%)			0.322^b^
	Yes	17 (77.3%)		14 (63.6%)			

When the change in NRS scores within the groups was analyzed, a statistically significant decrease was observed in the RFT group at one and six months compared to baseline (p<0.001). Similarly, in the PRF group, a decrease was observed at one and six months compared with baseline, and this change was statistically significant (p<0.001) (Table 2).

**Table 2:** Change in NRS scores NRS: numeric rating scale; SD: standard deviation; Test st: test statistic; Group RFT: trigeminal ganglion radiofrequency thermocoagulation group; Group PRF: maxillary or mandibular (max/mand) nerve pulsed radiofrequency group

		NRS basal	NRS 1 month	NRS 6 months	Test st.	P-value
Group RFT	Mean rank	2.82	1.23	1.95	35.086	<0.001
	Mean ±SD	7.91 ±0.75	2.73 ±2.64	2.73 ±2.64		
Group PRF	Mean Rank	2.82	1.25	1.93	34.696	<0.001
	Mean ±SD	7.82 ±0.73	3.55 ±2.61	4.41 ±2.13		

## Discussion

In the present study, we compared the efficacy of TG RFT and max/mand PRF in the management of TN. The results showed that although the NRS scores were lower for the TG RFT group at one and six months, the difference between the two groups was not statistically significant. Both treatments provided significant pain relief compared to baseline. In addition, there was no difference in the reduction of medication consumption after the treatments between the groups, as measured by MQS III at six months.

MVD is the preferred surgical procedure for classical TN when vascular compression of the trigeminal nerve is confirmed. However, despite its high success rate, MVD is associated with serious complications and a mortality rate of 0.2-0.5% [10,13]. RFT of the TG is a viable alternative in the absence of vascular compression. The mechanism of action of TG RFT neurotomy for TN involves the application of radiofrequency energy to create a lesion in the TG, which interrupts the transmission of pain signals along the trigeminal nerve [14]. The success rate of TG RFT in treating TN is reportedly high, with some studies indicating an initial pain relief rate of up to 92% [15]. However, the procedure is associated with complications such as dysesthesia, anesthesia dolorosa, and corneal numbness, and complication rates vary depending on the technique used and temperature settings during the procedure [14]. To avoid these risks, PRF has become increasingly common. However, the efficacy of PRF for max/mand branches has not yet been evaluated. In our study, a success rate of 63.6% (14/22) was observed with max/mand PRF at the six-month follow-up, while the TG RFT group had a success rate of 77.3% (17/22). Although the success rate was higher in the RFT group, no significant difference was found between the two groups.

PRF has emerged as a promising alternative to RFT. PRF is a minimally invasive procedure that offers significant advantages, primarily its non-destructive nature, which helps avoid complications commonly associated with conventional RFT [16,17]. PRF has a distinct and beneficial mechanism of action. It operates by delivering short bursts of radiofrequency energy, which leads to a temporary modulation of nerve function without causing permanent damage. This modulation is achieved through a decrease in pro-inflammatory cytokines, an increase in cytosolic calcium concentration, a general effect on the immune system, and a reduction in the formation of free radical molecules [18]. The ignition theory is one of the main mechanisms proposed in the pathophysiology of TN. Spontaneous ignitions in the afferent neurons cause hyperexcitability [3]. These spontaneous ignitions can be reduced by PRF treatment [19].

Clinically, PRF has been successfully applied to various neural structures, including the dorsal root ganglion, sphenopalatine ganglion, and suprascapular nerve. These applications have shown promising results in managing pain without causing significant tissue damage [20,21]. Moreover, the safety profile of PRF is noteworthy. Studies have reported no serious complications associated with its use, highlighting its suitability for patients in whom traditional interventions may pose a higher risk [22]. In our study, no complications were observed in the PRF group, whereas the following adverse events were observed in the RFT group: hypoesthesia of the upper jaw in one patient, hypoesthesia of the palate in one patient, and masseter muscle weakness in one patient. However, the longest duration of these adverse events was approximately one month, and they resolved spontaneously without treatment.

CT and FL-guided interventions are commonly employed in the management of TN, which unfortunately expose both patients and practitioners to ionizing radiation. Although the exact amount of radiation varies depending on the specific procedure and equipment used, there is a consensus that minimizing exposure is crucial for safety [23]. The use of US is becoming more prevalent as it offers a radiation-free alternative, with the added benefit of visualizing vascular structures, which is particularly advantageous in the vascular-rich regions of the head and face. This imaging modality may contribute to a reduction in procedural complications [24,25]. Recent studies have explored the efficacy of US in this context. Anugerah et al. [26] attempted a US-guided blockade of the pterygopalatine fossa (PPF) for the maxillary nerve. Nader et al. [27] successfully performed a US-guided maxillary block in 15 patients with TN, confirming the accuracy of the block using a pin-prick test.

Although the max/mand nerves are deeply situated and challenging to visualize directly with US, the identification of potential spaces between bony structures and eliciting sensory and motor responses with RF can increase the likelihood of nerve localization, as shown in our study. In addition, the potential gap between the CoP and the maxilla anterior to the CoP for visualization of the MA, as used in our study, may provide a wider bony window than the classic mandibular notch approach from posterior to anterior. In a study by Kampitak et al. [28] involving 10 fresh cadavers, they were able to reach the PPF in all cases by injection from the anterior aspect of the CoP. When they attempted to reach the PPF posteriorly through the mandibular notch between the CP and CoP, they detected methylene blue in the buccal fat pad more than in the PPF. In this study, an in-plane technique was used from anterior to posterior (AP). As it may be difficult to reach the maxillary nerve located anteriorly due to the posterior movement of the needle tip with the AP approach, we reached the maxillary nerve in all patients using an out-of-plane technique, which we also use in our routine clinical practice. We confirmed that the max/mand nerves were reached with RF stimulation and noted a positive response to the diagnostic block.

Although PRF and RFT treatments have high success rates and are relatively safe, they are costly and require well-trained personnel [29]. Hence, it may not be accessible in all conditions and countries. Peripheral blocks with local anesthetics have been used for this purpose, particularly at high concentrations (e.g., 10% lidocaine) [10]. The authors suggested that high concentrations of local anesthetics may be effective in the treatment of TN owing to their neurotoxic effects. However, a mean analgesic period of 7.5 months was achieved, which was found to be significantly shorter than that of RFT treatment. We were not able to follow up with our patients for the duration of the study, and our study had several limitations. Firstly, the follow-up period was limited to six months; therefore, we could not evaluate the longer mean pain-free period in both groups. Second, we could not evaluate the effectiveness of the interventional procedures on functionality. Third, because of the differences in the interventions and the use of US, the practitioner was not blinded, and it was a single-blind design with only the assessor blinded. Fourth, we were unable to assess the cervical degenerative myelopathic changes that may be involved in the pathophysiology of TN. The potential link between cervical degenerative disease and TN may be attributed to biomechanical changes or nerve root irritation that exacerbate trigeminal nerve sensitivity [30]. Understanding these relationships is crucial for developing targeted and effective treatment strategies.

## Conclusions

Based on our findings, while the RFT group experienced a higher incidence of procedure-related adverse events, these were not statistically significant and resolved spontaneously without the need for medical treatment. Both RFT and PRF treatments effectively provided pain relief at the six-month follow-up compared to baseline. Although the RFT group had a slightly higher success rate, the difference was not statistically significant. However, the PRF group had the distinct advantage of enabling the performance of US-guided max/mand PRF without exposing patients to radiation. In addition, we believe that the potential space between the maxilla and CoP from the anterior side of the CoP with an out-of-plane technique to reach the maxillary nerve under US guidance provides easier visualization of the MA and needle maneuvering than the posterior approach. We recommend more randomized controlled trials with larger sample sizes and extended follow-up periods to further substantiate these findings.

## References

[1] (2018). Headache Classification Committee of the International Headache Society (IHS): The International Classification of Headache Disorders, 3rd edition. Cephalalgia.

[2] Lambru G, Zakrzewska J, Matharu M (2021). Trigeminal neuralgia: a practical guide. Pract Neurol.

[3] Bharti N, Sujith J, Singla N, Panda NB, Bala I (2019). Radiofrequency thermoablation of the Gasserian ganglion versus the peripheral branches of the trigeminal nerve for treatment of trigeminal neuralgia: a randomized, control trial. Pain Physician.

[4] Chu EC, Rissardo JP (2024). Conservative management of trigeminal neuralgia and degenerative cervical myelopathy: a case report. Cureus.

[5] Montano N, Conforti G, Di Bonaventura R, Meglio M, Fernandez E, Papacci F (2015). Advances in diagnosis and treatment of trigeminal neuralgia. Ther Clin Risk Manag.

[6] Cheng JS, Lim DA, Chang EF, Barbaro NM (2014). A review of percutaneous treatments for trigeminal neuralgia. Neurosurgery.

[7] Erdine S, Ozyalcin NS, Cimen A, Celik M, Talu GK, Disci R (2007). Comparison of pulsed radiofrequency with conventional radiofrequency in the treatment of idiopathic trigeminal neuralgia. Eur J Pain.

[8] Hong K, Georgiades C (2010). Radiofrequency ablation: mechanism of action and devices. J Vasc Interv Radiol.

[9] Sam J, Catapano M, Sahni S, Ma F, Abd-Elsayed A, Visnjevac O (2021). Pulsed radiofrequency in interventional pain management: cellular and molecular mechanisms of action - an update and review. Pain Physician.

[10] Ertilav E, Aydın ON (2022). Evaluation of the effectiveness duration of peripheral blocks applied with high concentration local anesthetic and steroid in trigeminal neuralgia. Agri.

[11] Gallizzi M, Gagnon C, Harden RN, Stanos S, Khan A (2008). Medication Quantification Scale Version III: internal validation of detriment weights using a chronic pain population. Pain Pract.

[12] Elawamy A, Abdalla EEM, Shehata GA (2017). Effects of pulsed versus conventional versus combined radiofrequency for the treatment of trigeminal neuralgia: a prospective study. Pain Physician.

[13] Degn J, Brennum J (2010). Surgical treatment of trigeminal neuralgia. Results from the use of glycerol injection, microvascular decompression, and rhizotomia. Acta Neurochir (Wien).

[14] Wang Z, Wang Z, Li K, Su X, Du C, Tian Y (2022). Radiofrequency thermocoagulation for the treatment of trigeminal neuralgia. Exp Ther Med.

[15] Wang Z, Su X, Yu Y (2022). A review of literature and meta-analysis of one-puncture success rate in radiofrequency thermocoagulation with different guidance techniques for trigeminal neuralgia. Eur J Med Res.

[16] Jia Y, Cheng H, Shrestha N, Ren H, Zhao C, Feng K, Luo F (2023). Effectiveness and safety of high-voltage pulsed radiofrequency to treat patients with primary trigeminal neuralgia: a multicenter, randomized, double-blind, controlled study. J Headache Pain.

[17] Jia Y, Pan Y, Ren H, Ji N, Luo F (2018). Effectiveness and safety of high-voltage pulsed radiofrequency to treat patients with primary trigeminal neuralgia: a multicenter, randomized, double-blind, controlled study protocol. Pain Physician.

[18] Jorge DM, Huber SC, Rodrigues BL (2022). The mechanism of action between pulsed radiofrequency and orthobiologics: is there a synergistic effect?. Int J Mol Sci.

[19] Abd-Elsayed A, Martens JM, Fiala KJ, Izuogu A (2022). Pulsed radiofrequency for the treatment of trigeminal neuralgia. Curr Pain Headache Rep.

[20] Vuka I, Došenović S, Marciuš T, Ferhatović Hamzić L, Vučić K, Sapunar D, Puljak L (2020). Efficacy and safety of pulsed radiofrequency as a method of dorsal root ganglia stimulation for treatment of non-neuropathic pain: a systematic review. BMC Anesthesiol.

[21] Chua NH, Vissers KC, Sluijter ME (2011). Pulsed radiofrequency treatment in interventional pain management: mechanisms and potential indications-a review. Acta Neurochir (Wien).

[22] Pastrak M, Visnjevac O, Visnjevac T, Ma F, Abd-Elsayed A (2022). Safety of conventional and pulsed radiofrequency lesions of the dorsal root entry zone complex (DREZC) for interventional pain management: a systematic review. Pain Ther.

[23] Telischak NA, Heit JJ, Campos LW, Choudhri OA, Do HM, Qian X (2018). Fluoroscopic C-arm and CT-guided selective radiofrequency ablation for trigeminal and glossopharyngeal facial pain syndromes. Pain Med.

[24] Chong MS, Bahra A, Zakrzewska JM (2023). Guidelines for the management of trigeminal neuralgia. Cleve Clin J Med.

[25] Allam AE, Khalil AA, Eltawab BA, Wu WT, Chang KV (2018). Ultrasound-guided intervention for treatment of trigeminal neuralgia: an updated review of anatomy and techniques. Pain Res Manag.

[26] Anugerah A, Nguyen K, Nader A (2020). Technical considerations for approaches to the ultrasound-guided maxillary nerve block via the pterygopalatine fossa: a literature review. Reg Anesth Pain Med.

[27] Nader A, Kendall MC, De Oliveria GS, Chen JQ, Vanderby B, Rosenow JM, Bendok BR (2013). Ultrasound-guided trigeminal nerve block via the pterygopalatine fossa: an effective treatment for trigeminal neuralgia and atypical facial pain. Pain Physician.

[28] Kampitak W, Tansatit T, Shibata Y (2018). A cadaveric study of ultrasound-guided maxillary nerve block via the pterygopalatine fossa: a novel technique using the lateral pterygoid plate approach. Reg Anesth Pain Med.

[29] Costa N, Mounie M, Gombault-Datzenko E (2023). Cost analysis of radiofrequency ablation for adrenal adenoma in patients with primary aldosteronism and hypertension: results from the ADERADHTA pilot study and comparison with surgical adrenalectomy. Cardiovasc Intervent Radiol.

[30] Trager RJ, Theodorou EC, Chu ECP (2024). Association between trigeminal neuralgia and degenerative cervical myelopathy: a cross‐sectional study using US data. Neurol Clin Neurosci.

